# Microbiome-informed study of the mechanistic basis of methane inhibition by *Asparagopsis taxiformis* in dairy cattle

**DOI:** 10.1128/mbio.00782-24

**Published:** 2024-07-02

**Authors:** Nagaraju Indugu, Kapil Narayan, Hannah A. Stefenoni, Meagan L. Hennessy, Bonnie Vecchiarelli, Joseph S. Bender, Reeti Shah, Grace Dai, Satvik Garapati, Charles Yarish, Sergio C. Welchez, Susanna E. Räisänen, Derek Wasson, Camila Lage, Audino Melgar, Alexander N. Hristov, Dipti W. Pitta

**Affiliations:** 1 Department of Clinical Studies, New Bolton Center, School of Veterinary Medicine, University of Pennsylvania, Kennett Square, Pennsylvania, USA; 2 Department of Animal Science, The Pennsylvania State University, University Park, Pennsylvania, USA; 3 Department of Ecology and Evolutionary Biology, The University of Connecticut, Stamford, Connecticut, USA; University of Tennessee at Knoxville, Knoxville, Tennessee, USA

**Keywords:** hydrogenotrophic methanogens, methylotrophic methanogens, ruminal methanogenesis, methane mitigation

## Abstract

Copious amounts of methane, a major constituent of greenhouse gases currently driving climate change, are emitted by livestock, and efficient methods that curb such emissions are urgently needed to reduce global warming. When fed to cows, the red seaweed *Asparagopsis taxiformis* (AT) can reduce enteric methane emissions by up to 80%, but the achieved results can vary widely. Livestock produce methane as a byproduct of methanogenesis, which occurs during the breakdown of feed by microbes in the rumen. The ruminant microbiome is a diverse ecosystem comprising bacteria, protozoa, fungi, and archaea, and methanogenic archaea work synergistically with bacteria to produce methane. Here, we find that an effective reduction in methane emission by high-dose AT (0.5% dry matter intake) was associated with a reduction in methanol-utilizing *Methanosphaera* within the rumen, suggesting that they may play a greater role in methane formation than previously thought. However, a later spike in *Methanosphaera* suggested an acquired resistance, possibly via the reductive dehalogenation of bromoform. While we found that AT inhibition of methanogenesis indirectly impacted ruminal bacteria and fermentation pathways due to an increase in spared H_2_, we also found that an increase in butyrate synthesis was due to a direct effect of AT on butyrate-producing bacteria such as *Butyrivibrio*, *Moryella,* and *Eubacterium*. Together, our findings provide several novel insights into the impact of AT on both methane emissions and the microbiome, thereby elucidating additional pathways that may need to be targeted to maintain its inhibitory effects while preserving microbiome health and animal productivity.

**IMPORTANCE:**

Livestock emits copious quantities of methane, a major constituent of the greenhouse gases currently driving climate change. Methanogens within the bovine rumen produce methane during the breakdown of feed. While the red seaweed *Asparagopsis taxiformis* (AT) can significantly reduce methane emissions when fed to cows, its effects appear short-lived. This study revealed that the effective reduction of methane emissions by AT was accompanied by the near-total elimination of methane-generating *Methanosphaera*. However, *Methanosphaera* populations subsequently rebounded due to their ability to inactivate bromoform, a major inhibitor of methane formation found in AT. This study presents novel findings on the contribution of *Methanosphaera* to ruminal methanogenesis, the mode of action of AT, and the possibility for complementing different strategies to effectively curb methane emissions.

## INTRODUCTION

Global warming is driven by the release of greenhouse gases, with methane (CH_4_) being a great contributor. Indeed, the warming potency of CH_4_ is 80 times that of CO_2_ over a 20-year period, making it the second-largest contributor to global warming (1). The Paris Agreement of 2015 aimed to prevent a 1.5°C increase in global temperature from pre-industrial levels. However, 2023 marked the first time that such an increase has a greater-than-not probability of occurring within the next 5 years (2), suggesting that the proposed CH_4_ mitigation strategies, which thus far slow global warming by about 30% (3), are insufficient. As such, a focus on enteric CH_4_ emission from livestock, one of the largest contributors of anthropogenic CH_4_ emissions (4), is clearly warranted.

Livestock produces CH_4_ as a byproduct of methanogenesis, which occurs during the breakdown of feed in the rumen. The ruminant microbiome is a diverse ecosystem comprising billions of microbes, including bacteria, protozoa, fungi, and archaea. While bacteria facilitate carbohydrate breakdown, methanogenic archaea work synergistically with the bacteria, acting as a hydrogen (H_2_) sink and reducing bacterially produced substrates, ultimately resulting in methane production and emission (5). Depending on the substrates present in the feed, different methanogenesis pathways, including the hydrogenotrophic, methylotrophic, and acetoclastic pathways (6) are utilized within the rumen. While it is commonly believed that carbon dioxide (CO_2_)-utilizing methanogens like *Methanobrevibacter* are the dominant methane producers, recent studies demonstrated that methylotrophic methanogens, such as *Methanosphaera*, also play a large role in CH_4_ production (7, 8).

Although the distinct methanogenic pathways utilized in the rumen are each initiated by different enzymes, they all require methyl-coenzyme M reductase (MCR) for the final step of methanogenesis. Accordingly, livestock CH_4_-reduction technologies often include the addition of methanogenesis inhibitors into livestock feed, including the MCR inhibitor 3-nitrooxypropanol (3-NOP) (9, 10). In addition, supplementation with naturally occurring seaweeds such as *Asparagopsis taxiformis* (AT) (11) that contain halogenated CH_4_ analogs such as bromoform (CHBr_3_), capable of inhibiting the transfer of methyl groups and MCR catalytic activity (12, 13), has been gaining traction. However, the effects of AT appear unstable, potentially due to the loss of bromoform functionality over time (11), and its significant reduction of enteric CH_4_ emissions in dairy cattle is transient. Finally, while AT can significantly reduce methane-generating microbes such as *Methanomassilicoccaceae* and *Methanobrevibacter in vitro* (14), the consequential loss of a functional H_2_ sink that would accompany such inhibition could alter symbiotic relationships between microbes in the rumen. As the disruption in these critical symbiotic relationships could have a negative impact on feed intake and animal productivity (15), a comprehensive assessment of the effects of AT on the rumen microbiome is clearly warranted.

To determine if the gradual resistance to the anti-methanogenic effects of AT was due to alterations in rumen microbiome diversity and gene content, we utilized 16S rRNA, real-time PCR, and shotgun metagenomics analyses to identify the most common methanogenic species and the presence of genes encoding enzymes involved in the three predominant ruminal methanogenesis pathways. Moreover, we determined if alternative H_2_ sinks were engaged under conditions where methanogens were inhibited by AT and if AT had any direct impact on bacterial populations. Together, our results reveal an unexpected but significant contribution of methanol-utilizing *Methanosphera* toward ruminal methanogenesis, providing essential insight into the mechanistic basis of AT and the development of AT resistance mechanisms among ruminal methanogens. In addition, we identify both direct and indirect effects of AT on ruminal bacteria and fermentation pathways that could impact animal health and productivity and must therefore be considered before adopting such mitigation strategies.

## RESULTS

Twenty Holstein cows were randomly assigned to four [control, low 0.25% AT (LAT), high 0.5% AT (HAT), and oregano (O), another inhibitor of methanogenesis] treatments in a replicated 4 × 4 Latin square design in which each dairy cow was rotated between the four treatments in four periods. Toward the end of each period, enteric CH_4_ emissions were measured, and the results were published (13). The effects of AT supplementation at 0.5% of dietary dry matter (HAT) on dairy cattle in a Latin square design inhibited enteric CH_4_ emissions by 55% in periods 1 and 2, but the inhibitory effect of AT gradually declined by periods 3 and 4 (11). In contrast, LAT and oregano supplementation had no effect on enteric CH_4_ emissions in this study (11). To determine the effects of these treatments on the rumen microbiome, a source of enteric CH_4_ formation, ruminal samples collected using the stomach tube method during the prior study were isolated by filtering through three-layered cheesecloth, extracted for genomic DNA, and processed for 16S rRNA and metagenomics (metaG) in the current study.

### Sequencing information

To assess the diversity within the rumen archaeal community that could be contributing to methanogenesis, 930,776 raw partial 16S rRNA sequences were acquired from the rumen samples of 79 cows that were fed either a control diet (20), high AT (20), low AT (19), or oregano (20). Following rigorous quality filtering and denoising, 869,942 sequences were retained, with read counts per sample ranging from 2,490 to 20,134, identifying 192 unique amplicon sequence variants (ASV). Likewise, in the rumen bacterial community, 5,159,552 raw partial 16S rRNA sequences were obtained from the same 79 samples. After quality filtering and denoising procedures, 3,345,832 sequences were retained, with read counts per sample varying from 17,524 to 71,193, identifying 25,082 unique ASV. To assess AT-induced changes in microbiome function, we utilized shotgun metagenomic analysis. The use of the Illumina HiSeq platform generated 534,618,479 sequences across 60 samples. After quality filtering, approximately 11% of reads were removed, resulting in 473,192,284 high-quality reads, with sample sequences ranging from 3,756,824 to 12,019,902. Taxonomy assignment using Kraken2 revealed that approximately 96% of sequences were attributed to bacteria and roughly 2% each to archaea and eukaryotes. Notably, viruses constituted a minor fraction of the data set.

### Changes in methanogenic archaeal communities in response to AT supplementation

**Fig 1:**
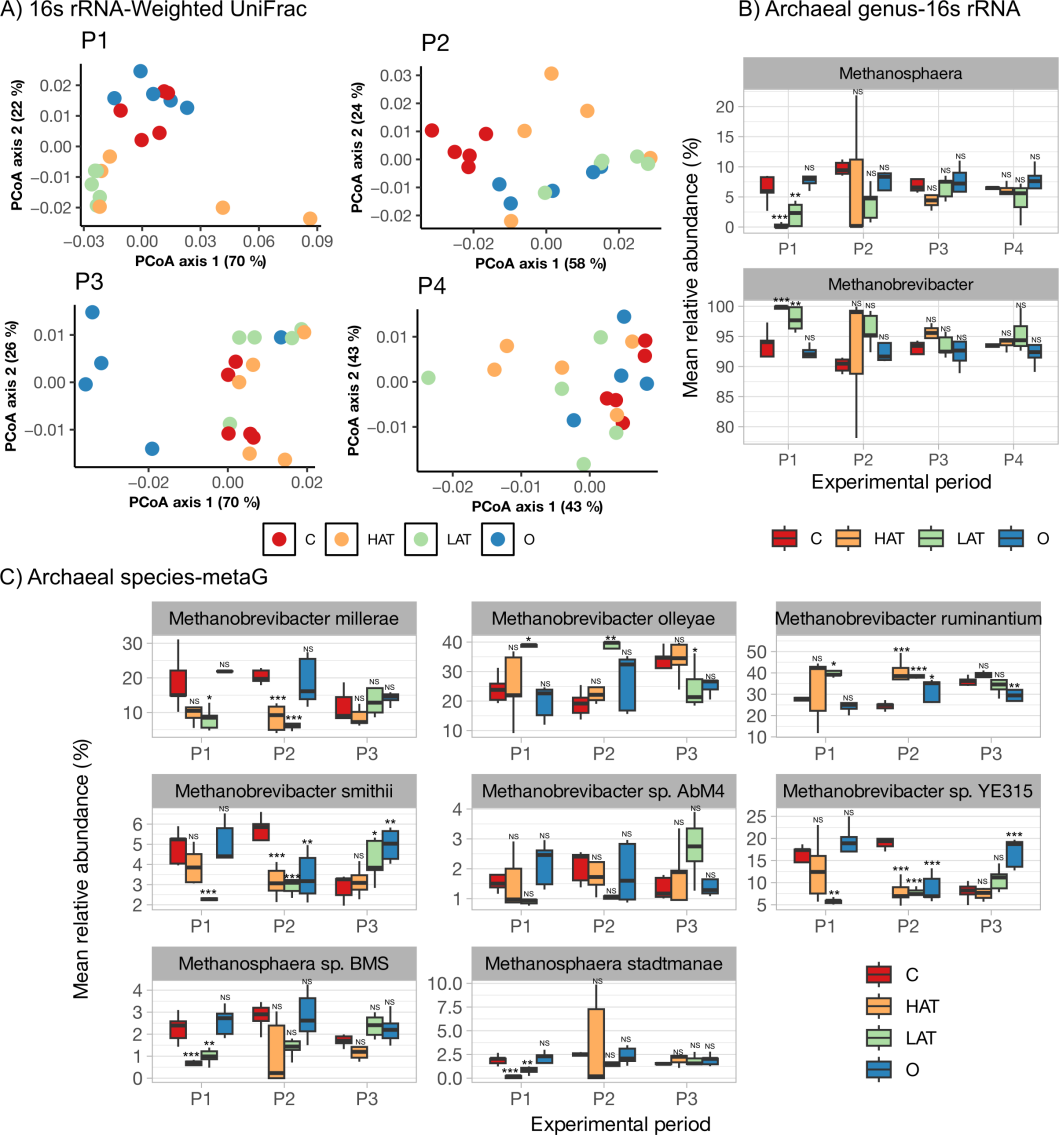
Assessment of rumen archaeal diversity and composition across different treatments by period. Treatments include control (C), HAT-, LAT-, and Oregano (O)-treated cows. (**A**) Principal coordinates analysis (PCoA) depicting weighted UniFrac distances of 16S rRNA archaeal compositions across periods 1, 2, 3, and 4. (**B**) Boxplots illustrating the abundance of the most prevalent archaeal genera based on 16S rRNA analysis. (**C**) Boxplots display the abundance of the most prevalent archaeal genera based on metagenomics analysis. Data significance is indicated as follows: NS, no statistical significance in generalized linear model; *P < 0.05; **P < 0.01; and ***P < 0.001.

To understand the mechanistic basis of AT (0.5% of dry matter intake) in curbing enteric CH_4_ emissions, we first investigated its effects on the ruminal methanogens that produce methane. First, methanogenic communities in the rumen of dairy cows over four experimental periods were compared at the community level (Fig. 1A), as assessed by the presence of commonly present populations (weighted UniFrac) and unique populations within each treatment (unweighted UniFrac; Fig. S1). Overall methanogenic communities differed by treatment, period, and their interaction in both the weighted and unweighted UniFrac analysis based on permutational multivariate analysis of variance (*P* < 0.05; PERMANOVA test, Table 1). Further pairwise comparisons revealed significant variations in community composition for certain combinations of treatments and periods (Table 1). In period 1, the LAT treatment exhibited a significant difference compared to the control group. Similarly, in period 2, the control group was significantly different from the HAT, LAT, and oregano groups. While oregano and LAT treatments displayed significant differences in community composition compared to the control group in period 3, no differences between treatments were noted in period 4. When considering the unweighted UniFrac matrix, significant differences were observed between the control and HAT groups in periods 1, 2, and 3, but the differences faded away by period 4. Thus, while large variations among commonly present populations by treatment and period interactions were observed, the less abundant methanogen populations were consistently reduced by HAT treatment during times at which reduced enteric CH_4_ emission was observed.

**TABLE 1:** PERMANOVA analysis for 16S rRNA archaeal amplicon sequencing data^*a*^

	Weighted		Unweighted	
	*R* ^2^	*P*-value	*R* ^2^	*P*-value
Overall				
Treatment	0.17	0.001	0.13	0.001
Period	0.07	0.018	0.07	0.004
Treatment: period	0.22	0.001	0.22	0.001
Period 1				
Control vs HAT	0.16	0.269	0.46	0.017
Control vs LAT	0.75	0.009	0.09	0.537
Control vs O	0.04	0.850	0.05	0.844
Period 2				
Control vs HAT	0.35	0.020	0.25	0.078
Control vs LAT	0.78	0.013	0.05	0.780
Control vs O	0.57	0.007	0.13	0.382
Period 3				
Control vs HAT	0.16	0.257	0.50	0.026
Control vs LAT	0.33	0.06	0.22	0.172
Control vs O	0.41	0.052	0.17	0.199
Period 4				
Control vs HAT	0.21	0.143	0.12	0.364
Control vs LAT	0.19	0.149	0.30	0.048
Control vs O	0.09	0.577	0.16	0.223

^*a*^ Treatment: control (C), HAT, LAT, and Oregano; period: P1, P2, P3, and P4. (.) *P* < 0.1; *P < 0.05; **P < 0.01; ***P < 0.001; *R*^2^: variance explained.

Second, we compared the taxonomy of individual methanogens using 16S rRNA sequencing analysis. We found that the archaeal genera *Methanobrevibacter* and *Methanosphaera* dominated the microbial community, collectively accounting for approximately 99% of the observed sequences (Fig. 1B). We observed a significant increase in the relative abundance of *Methanobrevibacter* in both the HAT and LAT treatment groups compared to the control during period 1 (*P* < 0.05). However, this difference became less pronounced in subsequent periods, with no statistically significant variations observed during period 2 or period 3 (*P* > 0.05). In contrast, the relative abundance of *Methanosphaera* decreased significantly in the HAT group during period 1 (*P* < 0.05), indicating a strong inhibitory effect of AT. However, during period 2, we observed a restoration of the relative abundance, although with considerable variation (*P* < 0.05). By period 3, there were no significant differences between treatments (*P* > 0.05), suggesting a potential adaptation to the high AT conditions. The Oregano treatment inhibited *Methanobrevibacter* but *Methanosphaera* was not affected in periods 1 and 2. When HAT was effective in inhibiting methanogenesis (in periods 1 and 2), methanol-utilizing *Methanosphaera* were almost completely eliminated, while no changes in *Methanobrevibacter* were noted. Interestingly, Oregano, which did not reduce CH_4_ in our study, did not alter *Methanosphaera* compared to control, further highlighting the correlation between decreased CH_4_ emissions and reductions in *Methanosphaera*.

Finally, we conducted a comprehensive analysis of the most common methanogenic species within *Methanobrevibacter* and *Methanosphaera* using shotgun metagenomics data (Fig. 1C). Among the *Methanobrevibacter* species, we identified six species, among which *Methanobrevibacter ruminantium*, *Methanobrevibacter ollyae*, and *Methanobrevibacter millerae* were the most dominant. *M. ruminantium* and *M. olleyae* showed a slight increase in the HAT group, although this increase did not reach statistical significance (*P* > 0.05). In contrast, the remaining four *Methanobrevibacter* species displayed a reduction, with *M. millerae* and *M.* YE315 showing significant (*P* < 0.05) decreases in cows in the HAT group. Furthermore, both *Methanosphaera* species exhibited reduced abundance in the HAT group during period 1, confirming the inhibitory effect of AT on these species. While 16S rRNA analyses revealed that hydrogenotrophic methanogens were minimally impacted or were increased by AT, the disappearance of *Methanosphaera* in the initial phase of the study again supported their importance in AT-associated reduction in enteric CH_4_ emissions. Consistent with this idea, prolonged feeding of AT beyond 5 weeks (into period 2) led to a rebounding effect in *Methanosphaera*, which was followed by the loss of AT function in reducing enteric CH_4_ emissions.

### Impact of AT on methanogenesis pathways

**Fig 2:**
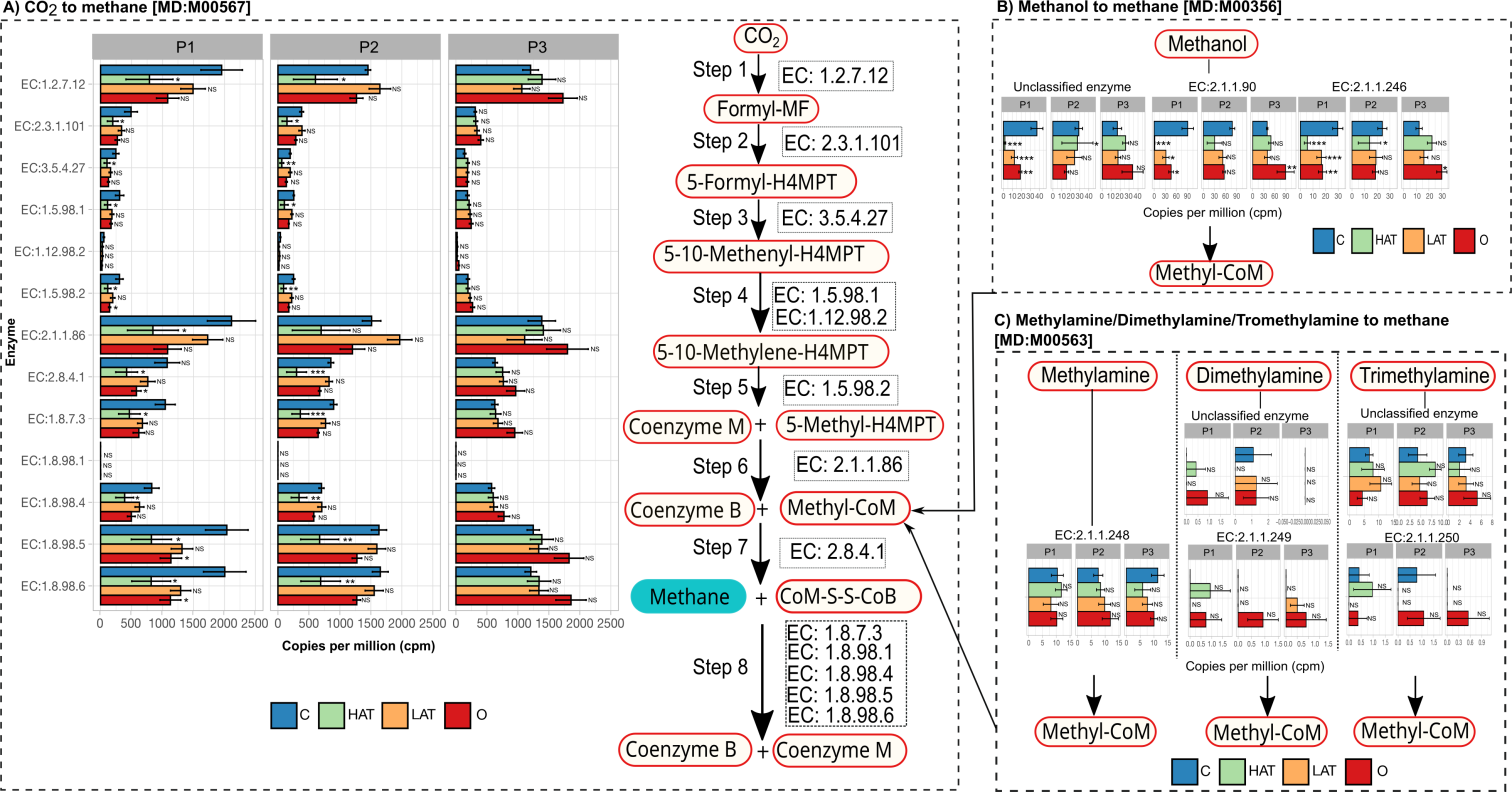
Comparison of gene abundance, measured in copies per million (CPM), associated with enzymes involved in different methanogenesis pathways across different treatments by period: control (C), HAT-, LAT-, and O-treated cows. (**A**) Carbon dioxide (CO_2_)/hydrogen (H2) methanogenesis pathway (Kyoto Encyclopedia of Genes and Genomes, KEGG pathway entry MD:M00567). (**B**) Methanol methanogenesis pathway (KEGG pathway entry MD:M00356). (**C**) Methylamine methanogenesis pathway (KEGG pathway entry MD:M00563). Data significance is denoted as follows: NS, no statistical significance in generalized linear model; *P < 0.05; **P < 0.01; and ***P < 0.001.

Using metagenomic shotgun data, we quantified the genes that code for enzymes involved in the three predominant ruminal methanogenesis pathways (CO_2_-, methanol-, and methylamine-reducing pathways) in cows with and without AT supplementation (Fig. 2). In addition, the taxonomy of the annotated genes was tracked to assess the role of individual methanogenic lineages in methanogenesis.

### CO_2_-reducing methanogenesis pathway

The CO_2_-reducing pathway catalyzes the conversion of CO_2_ and hydrogen gas (H_2_) into CH_4_. The CO_2_-reducing pathway encompasses eight steps (Fig. 2A), with steps 1–5 being unique to this pathway and steps 6–8 shared among all three methanogenic pathways. As we can successfully identify all the genes encoding the enzymes involved in the CO_2_-H_2_O pathway through metagenomic analysis, we next assessed the gene copy number of specific enzymes within this pathway across all treatments by periods (Fig. 2). Notably, the copy number of the gene encoding the enzyme EC: 1.2.7.12 (representing step 1 in Fig. 2A), which is involved in the reduction of CO_2_ to formylmethanofuran, exhibited the highest abundance among all the enzymes involved in steps 1–5. In periods 1 and 2, the gene copy number for this enzyme was significantly lower in the AT- and Oregano-supplemented cows compared to the control cows (*P* < 0.05 for both periods). However, in period 3, similar abundances were observed across all groups (*P* > 0.05), consistent with the loss of AT’s effect on methane inhibition. Additionally, the gene copy number for enzymes EC: 2.3.1.101 (step 2), 3.5.4.27 (step 3), 1.5.98.1 (step 4), and 1.5.98.2 (step 5) was significantly reduced (*P* < 0.05) in the AT- and Oregano-supplemented cows compared to the control cows in both periods 1 and 2; however, the differences between treatments were lost by period 3 where the effect of AT on enteric methane formation was lost.

*Methanobrevibacter* species predominantly associated with the CO_2_-reducing pathway (steps 1–5) (Dataset S1A), including *Methanobrevibacter* (*M. ruminantium* M1*, M. olleyae* YLM1*, M. millerae* SM9*, Methanobrevibacter* sp. YE315*, M. smithii* ATCC 35061*, and Methanobrevibacter.* sp. AbM4). Collectively, these six species accounted for approximately 93% of the genes encoding for enzymes involved in steps 1–5 of the CO_2_-reducing pathway. Notably, *M. ruminantium* M1 was the most abundant species, followed by the remaining five *Methanobrevibacter* species.

### Impact of AT on methanol- and methylamine-utilizing pathways

In the methanol-utilizing pathway (Fig. 2B), a notable difference was observed in the copy number of genes encoding the enzyme methanol-corrinoid protein co-methyltransferase (*mtaB*; EC: 2.1.1.90) when compared to the other two enzymes (*mtaA*; EC: 2.1.1.246 and Unclassified enzyme). The gene copy number of all three enzymes exhibited a significant reduction (*P* < 0.05) in HAT compared to control in period 1. However, all three enzymes appeared to increase in period 2 compared to period 1 in HAT samples, indicating a rebounding effect. While the unclassified genes and mtaB did not show any significant difference, the gene copy number for EC: 2.1.1.246 was still lower (*P* < 0.05) in HAT compared to control samples. By period 3, all genes rebounded, with no significant (*P* > 0.05) difference between the control and HAT groups.

Two species of *Methanosphaera* were predominantly associated with the methanol-utilizing pathway (Dataset S1B), with *Methanosphaera stadtmanae* DSM 3091 accounting for 39%, closely followed by *Methanosphaera* sp. BMS at 33%. Other contributors included methanogens such as *Methanobrevibacter smithii* ATCC 35061 (13%), *Methanogenic archaeon* ISO4-H5 (1%), and bacteria (2%). Collectively, the five archaea species represented approximately 93% of the identified methanol-utilizing archaea. A notable observation was the intriguing absence of the two *Methanosphaera* species, *Methanosphaera stadtmanae* DSM 3091 and *Methanosphaera* sp. BMS, within the HAT group during period 1, which is in agreement with the loss of genes coding for enzymes involved in the methanol pathway. However, as the two species began to rebound in period 2, this was also accompanied by changes in gene copy number in the methanol-utilizing pathway, with the latter still being lower in HAT compared to control. By period 3, the effect of HAT was lost completely, as *Methanosphaera* were completely restored, and no differences were noted in either *Methanosphaera* species or the genes involved in the methanol-utilizing pathway between control and HAT. Oregano did not have an effect on *Methanosphaera* or genes associated with the methanol pathway, suggesting the latter pathway was resistant to the effects of Oregano. A reduction in both *Methanosphaera* and methanol-utilizing genes is consistent with a reduction in CH4 emissions by HAT in periods 1 and 2. As Oregano had no effect on either CH4 emissions or *Methanosphaera* and its genes, our results suggest that *Methanosphaera* has a major role in ruminal methanogenesis.

In the methylamine-utilizing pathway (Fig. 2C), most of the genes coding for enzymes involved in the transfer of methylamines were found in greater copy number relative to those involved in the transfer of dimethyl or trimethylamines, although the total gene copy number was much lower than those involved in other pathways. Across all animals, copies of the gene coding for the enzyme methylamine-corrinoid protein co-methyltransferase (EC: 2.1.1.248) were negligible [<10 copies per million (CPM); mean]. No differences in gene copy number were noted between treatment groups, suggesting that the impact of AT on methylamine-utilizing methanogens was negligible.

### Impact of AT on methyl-coenzyme M reductase, the connecting point for all methanogenesis pathways

The enzyme MCR (EC: 2.8.4.1) plays a crucial role in CH_4_ formation by catalyzing the incorporation of methyl coenzyme M (Co-M) and coenzyme B (Co-B), resulting in the production of a heterodisulfide and the release of CH_4_ in the penultimate step (16). In this study, copies of the gene encoding MCR were found to be among the most abundant genes associated with methanogenesis pathways (Fig. 2A).

To compare the effects of LAT, HAT, and Oregano, the percent reduction in MCR gene copy number between the respective inhibitor and control was calculated (Table 2). These results revealed a significant decrease (*P* < 0.05) in the gene copy number of MCR in HAT compared to control, with reductions of 61% and 65% observed during periods 1 and 2, respectively. However, with the loss of AT’s effect on methane inhibition, the gene copy number of MCR was increased by 19% in HAT compared to control by period 3.

**TABLE 2:** Effect of *Asparagopsis taxiformis* on enteric methane yield, hydrogen emission, and genes (copies per million) encoding for methyl-coenzyme M reductase enzyme (EC: 2.8.4.1) in the rumen of dairy cows^*a*^

		Mean				% Change			Significance		
		C	HAT	LAT	O	HAT	LAT	O	C vs HAT	C vs LAT	C vs O
Period 1	CH_4_ (g)	338.84	128.07	349.16	409.40	62	−3	−20	0.001	0.809	0.113
	H_2_ (g)	1.34	10.26	3.62	1.05	−87	−63	22	<0.001	<0.001	0.347
	K00399	397.53	149.59	271.08	209.78	62	32	47	0.033	0.166	0.037
	K00401	324.57	125.84	231.44	157.26	61	29	52	0.050	0.254	0.042
	K00402	353.69	145.28	259.77	211.60	59	27	40	0.042	0.250	0.086
	EC:2.8.4.1	1,075.79	420.71	762.29	578.64	61	29	46	0.018	0.287	0.065
Period 2	CH_4_ (g)	410.24	132.97	354.93	336.47	68	13	18	<0.001	0.316	0.116
	H_2_ (g)	1.39	10.95	3.97	1.40	−87	−65	−1	<0.001	0.017	0.960
	K00399	326.00	111.45	296.23	248.87	66	9	24	0.006	0.273	0.002
	K00401	237.11	95.06	251.34	194.71	60	−6	18	0.032	0.604	0.031
	K00402	291.54	94.48	277.82	231.02	68	5	21	0.007	0.576	0.017
	EC:2.8.4.1	854.65	300.99	825.39	674.60	65	3	21	0.002	0.994	0.278
Period 3	CH_4_ (g)	362.98	336.33	339.74	390.58	7	6	-8	0.597	0.595	0.525
	H_2_ (g)	1.40	4.72	2.05	1.53	−70	−32	−9	0.006	0.014	0.628
	K00399	226.56	273.03	275.72	332.71	−21	−22	−47	0.345	0.069	0.051
	K00401	183.79	225.10	206.60	294.22	−22	−12	−60	0.313	0.457	0.058
	K00402	221.99	255.70	282.62	338.04	−15	−27	−52	0.368	0.077	0.085
	EC:2.8.4.1	632.35	753.83	764.95	964.97	−19	−21	−53	1.000	0.796	0.090

^*a*^ K00399, alpha subunit of MCR; K00401, beta subunit of MCR; K00402, gamma subunit of MCR; and C, control.

As MCR is composed of three subunits, alpha (K00399), beta (K00401), and gamma (K00402), we next looked to see if AT preferentially targeted one of the three subunits to inhibit methane formation. In period 1, all three subunits were inhibited to a similar extent by AT with reductions of 62%, 61%, and 59% of K00399 (*P* < 0.05), K00401 (*P* = 0.05), and K00402 (*P* < 0.05), respectively, compared to the control group. Notably in period 2, despite a noticeable rebounding effect of the methanol-utilizing pathway, the gene copy number of MCR enzyme continued to be inhibited (*P* < 0.05), with a reduction of 66%, 60%, and 68% in HAT-supplemented cows compared to control. In contrast, in period 3, the gene copy number of these subunits rebounded (*P* > 0.05) in the HAT-supplemented group and was numerically higher compared to the control group.

We also investigated the archaea species associated with MCR genes (Table 3). Among the identified species, a total of six *Methanobrevibacter* species, including *M. ruminantium* M1, *M. olleyae* YLM1, *M.* sp. YE315, *M. millerae* SM9, *M. smithii* ATCC 35061, and *M.* sp. AbM4, along with two *Methanosphaera* species, *M.* sp*.* BMS and *M. stadtmanae* DSM 3091, collectively made up approximately 93% of the total archaea associated with MCR. As indicated in Table 3, the species with the highest percent contribution to MCR were *Methanobrevibacter* species, particularly *M. ruminantium* M1*,* which was higher in HAT and LAT compared to the other two groups. *Methanosphaera* was negligible in period 1 but proportionally increased by period 2 although the contribution was insignificant based on gene copy number. Because the activity of MCR gene transcripts is several folds higher than the corresponding genes (17, 18), the MCR gene copy number may not be truly reflective of function. As such, the use of metatranscriptomics may shed more light on the contribution of individual methanogens to the MCR enzyme that catalyzes methane formation.

**TABLE 3:** Mean values of methyl-coenzyme M reductase enzyme (EC: 2.8.4.1) and the contribution of individual methanogenic archaea (copies per million) to MCR enzyme in the rumen of dairy cows under different treatments within each period

	Mean				Significance		
	C	HAT	LAT	O	C vs HAT	C vs LAT	C vs O
Period 1							
EC:2.8.4.1	1,076	420.7	762.3	578.6	0.022	0.160	0.049
*M. millerae* SM9	154.58	49.39	68.10	81.65	0.009	0.016	0.027
*M. olleyae* YLM1	198.48	95.49	174.92	106.65	0.078	0.598	0.078
*M. ruminantium* M1	308.06	155.91	322.15	154.41	0.084	0.851	0.084
*M. smithii* ATCC 35061	101.48	36.47	47.37	56.08	0.017	0.026	0.041
*M.* sp. AbM4	28.18	11.94	17.20	17.42	0.024	0.062	0.062
*M.* sp. YE315	153.38	41.30	70.23	89.04	0.001	0.007	0.021
*M.* sp. BMS	35.04	0.40	13.08	18.96	0.000	0.002	0.010
*M. stadtmanae* DSM 3091	20.67	0.29	7.79	11.53	0.000	0.002	0.015
Period 2							
EC:2.8.4.1	854.6	301	825.4	674.6	0.001	0.817	0.251
*M. millerae* SM9	124.41	29.46	76.49	72.68	0.000	0.004	0.004
*M. olleyae* YLM1	154.24	66.59	182.03	143.03	0.022	0.518	0.700
*M. ruminantium* M1	217.52	109.39	330.61	222.67	0.091	0.091	0.925
*M. smithii* ATCC 35061	86.02	20.56	53.80	53.97	0.000	0.006	0.006
*M.* sp*.* AbM4	27.57	9.05	18.45	19.15	0.001	0.051	0.051
*M.* sp. YE315	135.71	27.63	77.88	66.05	0.000	0.001	0.000
*M.* sp*.* BMS	26.58	6.77	21.72	32.42	0.052	0.525	0.525
M. *stadtmanae* DSM 3091	17.57	10.03	15.01	20.26	0.678	0.678	0.678
Period 3							
EC:2.8.4.1	632.3	753.8	765	964.97	0.389	0.389	0.082
*M. millerae* SM9	59.17	75.78	91.73	133.14	0.473	0.253	0.014
*M. olleyae* YLM1	144.89	170.96	170.08	176.75	0.383	0.383	0.383
*M. ruminantium* M1	235.98	274.49	240.98	273.17	0.681	0.919	0.681
*M. smithii* ATCC 35061	44.65	54.22	70.97	91.23	0.491	0.106	0.010
*M*. sp. AbM4	16.27	20.55	23.45	24.45	0.282	0.120	0.120
*M*. sp. YE315	63.01	75.39	81.85	140.12	0.519	0.496	0.002
*M*. sp. BMS	18.87	15.69	19.26	34.78	0.957	0.957	0.118
*M*. *stadtmanae* DSM 3091	11.70	18.21	14.06	20.52	0.145	0.530	0.088

### Hydrogenases regulating H_2_ production under normal and inhibited methanogenesis

The inhibition of methanogenesis by AT resulted in a notable sevenfold increase in gaseous H_2_ concentrations compared to the control, as reported by Stefenoni et al. (11) and Table 2, consistent with the loss of an H_2_ sink. Therefore, we sought to ascertain whether the dynamics in H_2_ concentrations following AT supplementation were associated with discernible changes in hydrogenase activity. Hydrogenases are metalloenzymes that play a pivotal role in converting H_2_ to 2[H] + 2e. They are broadly categorized into [FeFe], which plays a role in sensing H_2_ concentrations and H_2_ production; [NiFe], which facilitates H_2_ uptake; and [Fe], the function of which remains currently unknown. Within the [FeFe] category, enzymes are further differentiated into A1–A4, B, and C1–C3 groups, with the former two groups regulating H_2_ production and the latter sensing H_2_ concentrations.

Our findings revealed that, within the scope of this study (Fig. S2), the predominant [FeFe] hydrogenases included [FeFe]A1, [FeFe]A3, [FeFe]B, and [FeFe]C, whereas the majority of [NiFe] hydrogenases included [NiFe]3a, [NiFe]3c, [NiFe]4d, and [NiFe]4e. The copy number of hydrogenase genes remained relatively consistent across the treatment groups throughout all three periods. The only notable numerical fluctuations observed in specific groups of hydrogenases included reduced copy numbers of [FeFe]A3, [NiFe]3a, [NiFe]3c, and [NiFe]4d and increased copy number of [NiFe]4a in the HAT group compared to the control in period 1. These data indicate that bifurcating hydrogenases [FeFe]A3 were inhibited in response to inhibited methanogenesis by HAT, and the spared H_2_ was sensed by an increase in sensory hydrogenases such as [FeFe]B in the HAT treatment compared to other treatments, in both periods 1 and 2. However, the effect was lost by period 3. A reduction in H_2_-utilizing hydrogenases such as [NiFe]3a, 3c, and 4d, which are abundant in CO_2_-reducing *Methanobrevibacter* and *Methanosphaera*, is expected under conditions of inhibited methanogenesis. Noteworthy is the increase in copy number of [NiFe]4a in HAT compared to control, as this group of hydrogenases is associated with formate reduction. Whether this formate is fermented by bacteria or utilized by methanogens remains unknown.

### Alternative H_2_ sinks under inhibited methanogenesis

We next determined if the H_2_ produced during AT-mediated inhibition of methanogenesis was redirected toward alternative H_2_ sinks (Dataset S2), potentially engaging in direct or indirect competition with methanogens. Methanogenesis emerged as the primary H_2_ sink, demonstrating a significant reduction (*P* < 0.05) in the HAT-treated group during period 1 and a numerical decrease in period 2, with no observable differences in period 3. In addition, respiratory hydrogenases and bifurcating hydrogenases exhibited a notable decrease (*P* < 0.05) in the HAT-treated groups during periods 1 and 2, although this trend was not evident in period 3 (*P* > 0.05). Alternative and sensory hydrogenases, along with cofactor-coupled bidirectional hydrogenases, showed a numerical tendency to decrease in the HAT group during periods 1 and 2, with no substantial change in period 3. Energy-converting hydrogenases, fermentative hydrogenases, and sensory hydrogenases displayed relatively consistent patterns across the study periods. These data imply that none of the recognized alternative H_2_ sinks increased under conditions in which methanogenesis was inhibited by HAT. However, the total hydrogenases were greatly reduced in HAT compared to control and Oregano in periods 1 and 2, indicating that H_2_ metabolism was perturbed in HAT treatments. Application of metatranscriptomics may shed more insights into the functionality of different H_2_ sinks and how H_2_ is regulated under inhibited methanogenesis by AT.

### Impact of AT on bacterial populations

Because bacteria-methanogen interactions are fundamental to the integrity and functionality of the rumen microbiome, and AT inhibited specific groups of methanogens more than others, we hypothesized that AT may have both direct and indirect effects (mediated via spared H_2_ under inhibited methanogenesis by AT) on rumen bacteria and fermentation pathways leading to differences in volatile fatty acid (VFA) production. At the phylogeny level, 16S rRNA sequencing analysis across all samples revealed that the dominant bacterial phyla were Firmicutes, Bacteroidetes, and Actinobacteria, collectively accounting for 96% of the bacterial community (Dataset S3A). Within the Firmicutes phylum, the unclassified *Clostridiales* (1 and 2) *Butyrivibrio* were the most abundant, constituting more than 36% of the community. Among the Bacteroidetes phylum, the genus *Prevotella* emerged as the most dominant, representing 18% of the community (Dataset S3B). To compare the taxonomic composition across all cows, a total of 82 bacterial taxa were examined, each with an abundance threshold of at least 0.1% in one or more samples. Interestingly, 18 bacterial taxa exhibited significant differences (*P* < 0.05; Proc Mixed) in abundance between AT-supplemented cows and the control group (Dataset S3B). Among these identified taxa, nine showed an exclusive treatment effect, while three demonstrated a combined effect of treatment and period interaction. Additionally, three taxa were influenced by both treatment and period interactions. Notably, these included three taxa, namely *Butyrivibrio*, unclassified *Eubacterium*, and *Moryella*, known for producing the short-chain fatty acid butyrate, all of which were more abundant in cows treated with HAT compared to the control group. While shifts in bacteria following the inhibition of methanogenesis noted by period interactions with treatments were expected, the direct effects of HAT on bacteria even after the loss of its inhibitory effects on CH_4_ production are noteworthy and warrant further investigation.

### Effect of AT on fermentation pathways

**Fig 3:**
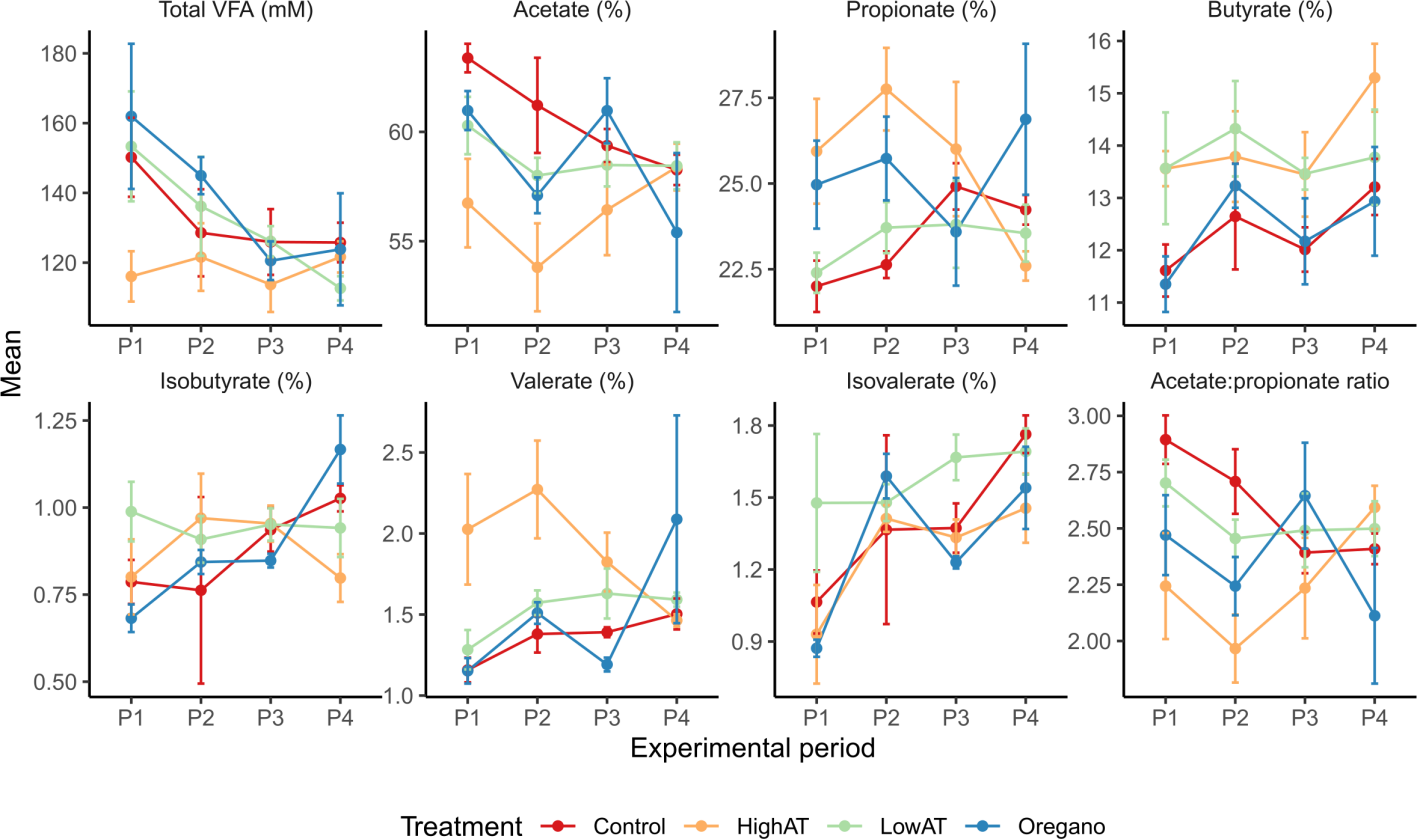
Effect of *Asparagopsis taxiformis* on rumen pH and VFA in lactating dairy cows across different treatments by period.

As we hypothesized that H_2_ spared under inhibited methanogenesis by AT would directly and indirectly impact bacterial fermentation, we next explored variations in fermentation pathways that contribute to VFA production by tracking changes in total and individual VFA pathways and the bacteria with which they were associated across treatments and periods (Fig. 3; Fig. S3). Surprisingly, concentrations of total VFA were consistently lower in HAT compared to all other treatment groups throughout the study, indicating that the effects of HAT on VFA production were not dictated by the degree of inhibition of methanogenesis. Overall, the molar proportion of acetate was reduced and those of propionate, butyrate, and valerate increased (*P* < 0.05) in HAT compared to control across all periods. To determine which bacteria might be contributing to VFA production, we initially conducted a correlation analysis between the molar proportions of individual VFA and bacterial populations identified through 16S rRNA sequencing across all samples. Bacterial taxa exhibiting significant differences (*P* < 0.05) between the control and AT-treated groups across all sampling periods were selected for correlation analysis with fermentation parameters. Our findings revealed a positive association between specific bacterial genera from the Firmicutes phylum, such as *Eubacterium*, *Moryella*, and *Butyrivibrio*, and the proportions of butyrate and valerate (Fig. S3), which were significantly increased in HAT treatment compared to other treatments across all periods.

**Fig 4:**
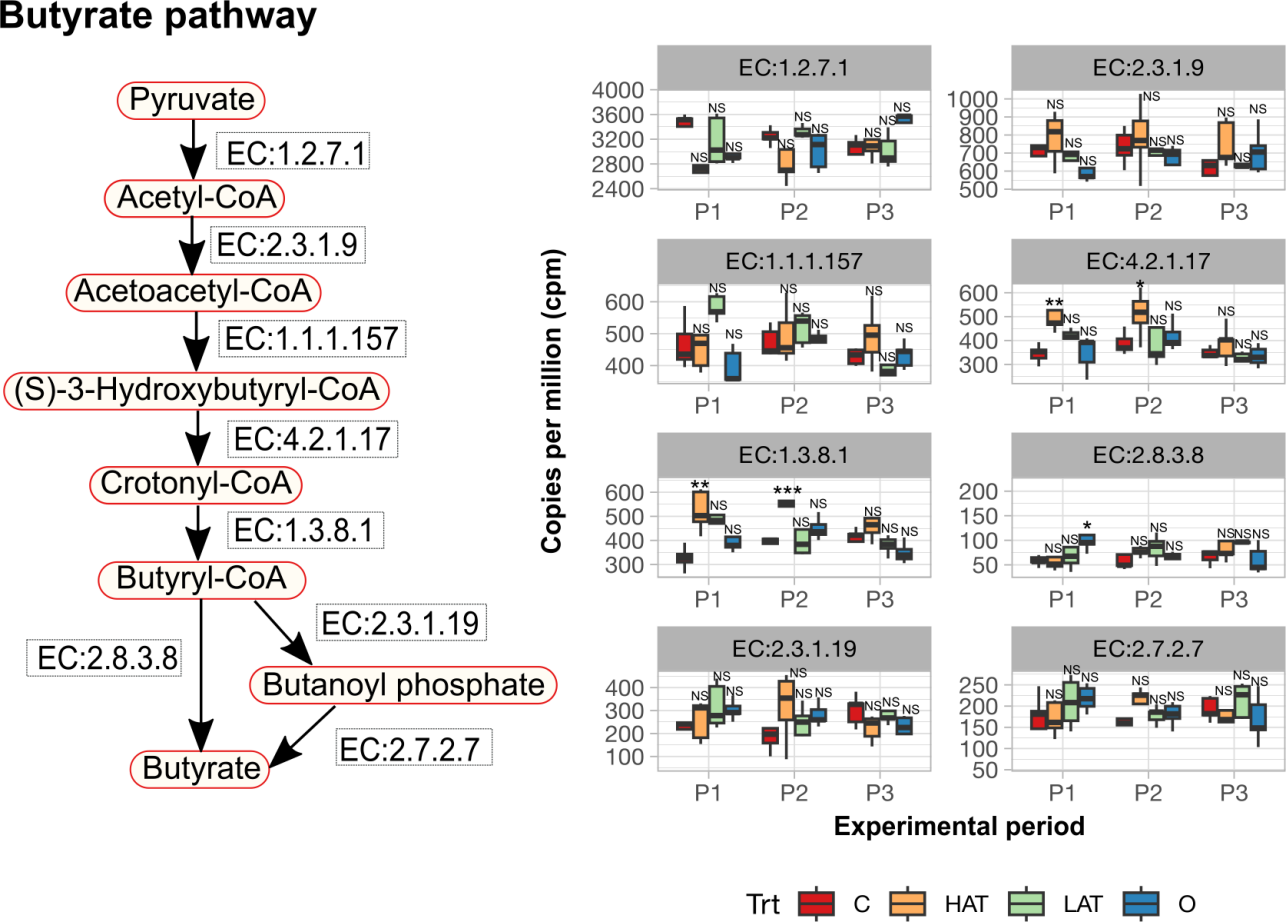
Comparison of gene abundance, measured in copies per million, associated with enzymes involved in butyrate pathway between different treatments by period: control (C), HAT-, LAT-, and Oregano (O)-treated cows. Data significance is denoted as follows: NS, no statistical significance in generalized linear model); *P < 0.05; **P < 0.01; and ***P < 0.001.

Subsequently, we investigated the presence of genes encoding enzymes involved in the distinct pathways leading to butyrate formation (Fig. 4). Noteworthy disparities in gene copy number were observed between the control and HAT treatment groups for enzymes participating in the butyrate pathway. Particularly, genes encoding the enzyme EC: 1.3.8.1, responsible for the conversion of crotonyl-CoA to butyryl-CoA, exhibited a significant increase (*P* < 0.05) in HAT samples compared to the control group in periods 1 and 2. These findings align with the observed increase (*P* < 0.05) in the molar proportions of butyrate in HAT samples compared to the control group (Fig. 3). The copy number of genes encoding the enzyme EC:2.8.3.8, which is responsible for the conversion of butyryl-CoA to butyrate (BP1 pathway), and EC:2.3.1.19 and EC:2.7.2.7, contributing to the conversion of butyryl-CoA to butyrate via the butanoyl phosphate (BP2 pathway), demonstrated numerical increases in the HAT treatment group compared to the control. However, these differences did not reach statistical significance. Identified bacteria associated with the butyrate pathway are detailed in Dataset S1F.

### Interactions within the rumen microbiome in different periods

As the effect of HAT on CH_4_ inhibition was noted in periods 1 and 2, but faded away by periods 3 and 4 and was accompanied by similar responses in *Methanosphaera* (except in period 2) as well as both direct and indirect effects on bacterial populations, we next performed a correlation analysis by period (Fig. S4A). As expected, CH_4_ emission was negatively correlated with H_2_ concentrations, propionate, and valerate, consistent with a negative correlation with rapid fermenting bacteria such as *Sharpea, Butyrivibrio, Lactobacilli, Moryella*, and *Eubacterium* and positively correlated with acetate and *Ruminococcaceae* bacterial lineages. As CH_4_ was negatively correlated with H_2_, the responses of H_2_ were opposite to those observed for CH_4_. Specifically, the most abundant methanogen *Methanobrevibacter* showed a positive correlation with H_2_, butyrate, and valerate and negative associations with acetate and all bacteria associated with acetate, while *Methanosphaera* showed the opposite pattern. Within the abundant bacteria, there were both positive and negative associations observed in period 1. While the associations noted in period 1 were expected (with both normal and inhibited methanogenesis samples included), these patterns were completely lost in period 2 (Fig. S4B). None of the bacteria seemed to correlate among themselves or with either VFA or CH_4_ emissions. This could be attributed to carryover effects of the previous treatments, plus possible adaptation mechanisms in AT treatments. Notably, in period 3 (Fig. S4C), when the effects of AT are lost (normal methanogenesis across all treatments), the rumen microbiome appeared to restore to normalcy, with CH_4_ emissions positively correlated with acetate and *Methanosphaera* and negatively correlated with propionate, butyrate, valerate, and *Methanobrevibacter*. Both positive and negative associations among bacterial lineages were noted, signifying the normal function of the rumen microbiota. In period 4 (Fig. S4D), much more intense interactions among bacteria were noted, with only limited interactions with methanogens. Finally, as there were 20 animals including first and 2+ lactation cows, the cow-to-cow variation was notable as shown in the sequence plot (Fig. S5) for CH_4_ emissions, changes in methanogens, and the MCR enzyme. With only five animals per treatment, and 20 animals rotated among four treatments in four periods with 28 days per period, some of the significant findings, particularly, in period 2, could not be substantiated. Overall, the positive association between CH_4_ emissions and *Methanosphaera* and MCR enzyme was pronounced in periods 1 and 3, showing that the inhibition of CH_4_ is associated with near elimination of *Methanosphaera*, which was reversed in period 3.

### Effect of AT on methanogenic isolates

**Fig 5:**
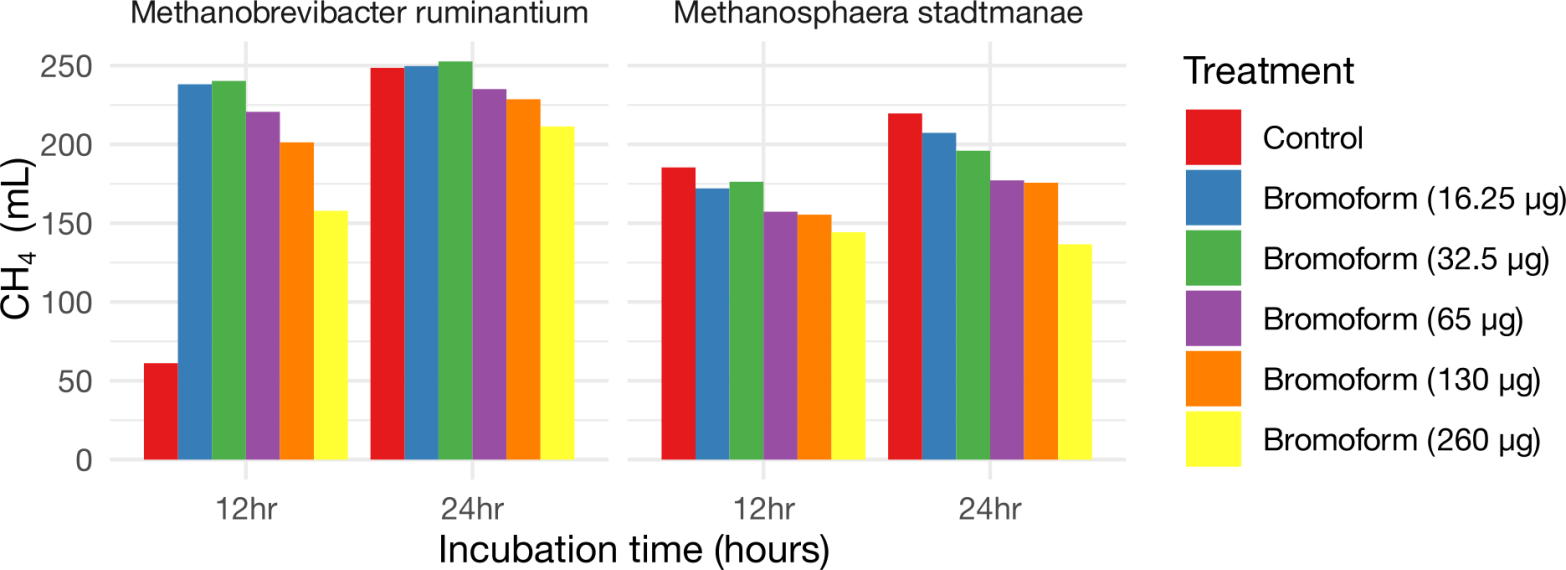
Effect of different Bromoform concentrations (0,16.25, 32.5, 65, 130, and 260 µg) added to two activated methanogenic isolates (*Methanobrevibacter ruminantium* and *Methanosphaera stadtmanae*) on methane yield at 12 and 24 h of incubation.

While it was evident from the metagenomic data that AT was selective for *Methanosphaera*, with *Methanobrevibacter* either showing no change or increased abundance, we next sought to validate our findings using pure methanogenic isolates. In our previous study (K. S. Narayan, A. C. B. Johnson, N. Indugu, J. Bender, H. A. Stefenoni, A. N. Hristov, A. Melgar, and D. Pitta, unpublished data), we assessed the methane-emitting potential of two pure isolates: CO_2_-utilizing *Methanobrevibacter ruminantium MI* and methanol-utilizing *Methanosphaera stadtmanae*. We reported that *Methanosphaera stadtmanae* makes three times more CH_4_ and grows faster (<12 h) than does *M. ruminantium* M1, which grows between 12 and 24 h in anaerobic Hungate tubes under laboratory conditions. To test the effect of bromoform, we added bromoform at both lower and higher concentrations than the concentration of bromoform reported in AT (approximately 65 µg/mL of media). Notably, we found that following the addition of bromoform up to 65 µg/mL, *Methanobrevibacter ruminantium* M1 reached its maximum CH_4_-emitting potential within 12 h, with only a marginal increase between 12 and 24 h. In contrast, in control Hungate tubes of *Methanobrevibacter ruminantium* M1 lacking bromoform, CH_4_ emission was lower at 12 h but reached its maximum by 24 h. These data reveal that the addition of bromoform did not affect the total CH_4_ emissions from *Methanobrevibacter ruminantium* M1, but instead accelerated its production (Fig. 5). Interestingly, increasing the dose of bromoform added to *Methanobrevibacter ruminantium* M1 culture tubes to 130 and 260 µg/mL, resulted in the inhibition of methane emission by 16% and 34%, respectively, at 12 h. This reduction was further reduced by 50% when measured at 24 h, revealing that the rapid changes that *Methanobrevibacter ruminantium* M1 undergoes when exposed to bromoform are only transient. The interaction between bromoform and individual ruminal isolates have not been previously described and hence the mechanistic basis remains unknown. In contrast, methane emissions from *M. stadtmanae* were gradually inhibited with increasing the dose of bromoform from 7% to 22% at 12 h and 6% to 40% at 24 h compared to control. These data clearly support findings derived from omic analysis that AT has selective preference for inhibiting *Methanosphaera*. Because the mechanisms of AT on individual methanogenic isolates are unknown, and HAT supplementation (dose at 65 µg/mL) resulted in a near elimination of *Methanosphaera*, other active compounds within AT that may be capable of suppressing *Methanosphaera* may exist. These data highlight the need to further investigate the mechanistic basis between AT and methanogen interactions to determine the role of *Methanosphaera* in ruminal methanogenesis and the mechanistic impact of AT on *Methanosphaera*.

## DISCUSSION

Enteric CH_4_ formation results from energy-inefficient fermentation of feed by microbiota within the bovine rumen. To reduce the contribution of enteric CH_4_ emission to global warming, seaweeds such as AT that are predicted to knock out methanogens have been utilized. However, the effects of AT appear transient, and bromoform, the active ingredient that suppresses CH_4_ formation, appears to lose its functionality over time (11). To gain a deeper understanding of the mechanistic basis underlying the effective yet transient effects of AT as a CH_4_ inhibitor, we undertook a comprehensive assessment of AT-dependent alterations in rumen microbiome diversity and gene content. Moreover, as AT is likely to interfere with symbiotic interactions between H_2_-producing microbes and H_2_-utilizing methanogens, we identified both the direct as well as indirect effects of AT (via spared H_2_ concentrations under inhibited methanogenesis) on rumen microbiota. Together, our findings provide mechanistic insight into AT-driven reduction in methane emissions and elucidation of additional pathways that may need to be targeted to maintain its inhibitory effects, while preserving microbiome health and animal productivity.

During period of AT-inhibited methanogenesis, methanol-utilizing *Methanosphaera* were almost completely eliminated, while only limited inhibitory effects were noted on ruminal hydrogenotrophic and methylamine-utilizing methanogens. Selective inhibition of *Methanosphaera* by HAT raises two important questions: first, what is the mechanistic basis underlying the effect of AT on *Methanosphaera*? Second, does the association between reduced CH_4_ emissions and the specific elimination of *Methanosphaera* in HAT-fed cows suggest that *Methanosphaera* has a greater role in enteric methane formation than previously thought? AT contains a number of halogenated CH_4_ analog components, including bromoform that likely inhibits CH_4_ formation by interfering with the transfer of methyl groups by methyl transferases and MCR during Wolfe’s cycle of methanogenesis. Vitamin B12 forms the central core of methyltransferases that transfer methyl group from methanol/methyl amines to coenzyme M, and bromoform likely inhibits methylotrophic methanogens via interfering with vitamin B12 synthesis. However, as diverse groups of methyl transferases are also present in hydrogenotrophic as well as methylamine-utilizing methanogens that are resistant to the effects of AT, it suggests additional, as yet unknown, mechanisms underly the inhibitory effects of AT on CH_4_ formation. In support, increasing the dose of bromoform to an activated culture of *Methanosphaera stadtmanae* did not completely inhibit methanogenesis, suggesting that additional compounds contribute to the selective preference of AT for *Methanosphaera*.

Enteric CH_4_ emissions significantly increase 2–4 h following feed intake in dairy cows (19), and we reported that *Methanosphaera* lineages dominate during this time when compared to *Methanobrevibacter* lineages (20), further supporting our finding that higher CH_4_ formation is strongly associated with an increase in the abundance of *Methanosphaera*. In prior studies (18), we reported that the metabolic activity of *Methanosphaera*, as revealed by metatranscriptomics, is approximately fivefold higher than what was estimated from the gene content of *Methanosphaera*ound using metagenomics. In a report (Narayan et al., unpublished), we found that a pure isolate of *Methanosphaera stadtmanae* grows faster (12 vs 24 h) and produces three times more CH_4_ than the same cell mass of *Methanobrevibacter ruminantium*. Furthermore, we (18) reported that hydrogenotrophic methanogens, including *Methanobrevibacter ruminantium,* were more sensitive to the MCR inhibitor 3-NOP (an analog of methyl coenzyme M) than were methylotrophic methanogens, resulting in a 30% reduction in total CH_4_ emissions (21). These data, in combination with our new data revealing that AT inhibition of enteric CH_4_ emissions by >50% is accompanied by the elimination of methanol-utilizing *Methanosphaera* with limited effect on other methanogens, suggest that *Methanosphaera* may have a greater share in total CH_4_ formation than previously thought. These new findings lay the foundation for targeting novel mitigation strategies and identifying functionally distinct complementary inhibitors that will help curb enteric CH_4_ formation.

Notably, our findings provide some insight into the transient and variable effects of AT on CH_4_ emission. Specifically, in period 2, when inhibition of CH_4_ emission by HAT was at 55%, *Methanosphaera* more than doubled (in absolute sequence numbers based on 16S rRNA sequence reads, and also metagenomic reads; Dataset S4) compared to control samples. Increased copy number of *Methanosphaera* to variable extents may indicate the onset of resistance mechanisms in response to AT. Halogenated compounds are either metabolized or excreted in the gastrointestinal tract of animals (22, 23), and methanogens are specifically able to “dehalogenate” compounds, with bromoform being dehalogenated faster than chloroform (24). Moreover, both coenzyme M methyltransferases and MCR can carry out reductive dehalogenation (25). Thus, the observed doubling of *Methanosphaera* by the end of period 2 may reflect the development of AT resistance through increased capacity to dehalogenate bromoform. Although bromoform concentrations in the rumen were not measured, milk collected at the end of periods 1 and 2 had higher concentrations of bromide, which forms following the dehalogenation of bromoform. However, it remains to be determined if the dehalogenation of bromoform by methanogens as speculated here or the sudden decrease of bromoform concentrations in seaweed as described in Stefenoni et al. (11) better explains the decrease of bromide in milk and may require more frequent sampling to help understand methanogen-bromoform interactions in the rumen. Furthermore, there may be other halogenated compounds such as iodoform that are as potent or more potent than bromoform for inhibiting methanogens. Regardless, *Methanosphaera* appears to have evolved mechanisms that help it overcome the inhibitory effects of AT, as reflected by their increased abundance toward the end of period 2.

In response to methane inhibition in HAT-supplemented cows, Stefenoni et al. (11) reported that H_2_ concentrations were three- to sixfold higher in treated cows. Surprisingly, we observed a decrease in both A1 (reduced-ferredoxin dependent hydrogenases) and A3 (bifurcating) hydrogenases in HAT compared to control. While such findings are not consistent with an increase in H_2_ gas, the concentration of dissolved H_2_ was not measured in this study. Previously, we reported an increase in dissolved H_2_ concentration in cows that were supplemented with the MCR inhibitor, 3-NOP. This increase was accompanied by a decrease in A3 bifurcating [FeFe] hydrogenases, with a concomitant increase in A1 ferredoxin only [FeFe] hydrogenases. We also observed a significant increase in group C3 hydrogenases, suggesting that increased H_2_ concentrations were sensed by H_2_-producing bacteria, that then switched their hydrogenases from A3 (bifurcating) to A1 (ferrdoxin-dependent). Such findings are in line with spared H_2_ increasing the partial pressure of H_2_. Because the 3-NOP experiment was a continuous study, changes in hydrogenases followed the flux in spared H_2_ concentrations under inhibited methanogenesis by 3-NOP over a 15-week period. Our inability to associate changes in free H_2_ gas with shifts in hydrogenases in the current study may be due to our rotating Latin square experimental design in which different animals were assigned to HAT in each period. Because the rumen microbiome differs between cows, it is reasonable to expect variations in hydrogenases, making it more difficult to detect patterns. It may also be that the measurement of dissolved H_2_ correlates with hydrogenases rather than free H_2_ gas concentrations. Finally, cows that were assigned to HAT treatment had lower dry matter intake and lower milk production, potentially explaining why we saw a decrease in both A1 and A3 hydrogenases, which reflect an overall reduction in fermentation patterns. As all of these issues could contribute to our inability to detect associations, continuous experiments for prolonged periods of feeding AT will be required to provide a mechanistic basis in connecting methane mitigation, spared H_2_, hydrogenases, and shifts in fermenting microbes in the rumen. Finally, as *Methanosphaera* is inhibited to a greater extent than is *Methanobrevibacter* in periods 1 and 2, it is possible that only a small degree of H_2_ is spared (only 1 mole of H_2_ is needed to reduce methanol), which agrees with the changes noted in hydrogenases. In addition, it is possible that AT directly impacts hydrogenotrophic bacteria to spare H_2_ in addition to inhibiting methanogens. Complementing metagenomic data with gene expression of hydrogenases using metatranscriptomics may provide a better picture of the link between hydrogenases and actual H_2_ production.

In addition to the expected indirect impact of AT on bacteria within the microbiota, we identified some direct effects that were less expected. Specifically, our bacterial analysis revealed 18 genera that were significantly increased in HAT-treated animals compared to control, even when the inhibitory effect of AT on methanogenesis was lost. Some of these bacteria include *Butyrivibrio*, *Eubacterium,* and *Roseburia* species, which belong to *Clostriales* XIV cluster, all known butyrate producers. Stefenoni et al. (11) previously reported that butyrate was significantly increased in HAT treatments compared to control. Butyrate synthesis involves the condensation of two acetate to acetoacetate and finally, acetoacetylcoA, which is then converted to crotonyl coA. A critical step in butyrate synthesis is the conversion of crotonyl-CoA to butyryl-CoA, which is catalyzed by a flavin-based electron bifurcation complex (butyryl-CoA dehydrogenase/electron-transferring flavoprotein complex; BcdA–EtfBC) (26). This step is irreversible and may not allow H_2_-forming butyrate producers to switch fermentation patterns. In the current study, butyrate production was consistently increased, whereas acetate to propionate ratio decreased (11) with a concomitant increase in gene copies of butyryl-CoA dehydrogenase, suggesting that butyrate synthesis may be an alternate sink to inhibited methanogenesis by AT. In the terminal step of the butyrate synthesis pathway, the conversion of butyryl-CoA to butyrate can occur via two distinct pathways, one mediated via the butyrate kinase pathway (BP1) and is mostly observed in *Clostridia* and the other mediated via the butyryl-CoA: acetate-CoA-transferase pathway (BP2) predominant in Negativicutes (27). As we found that HAT specifically increased the genes coding for the enzyme phosphate butyryltransferase (EC 2.3.1.19) (in periods 1 and 2 with no differences in periods 3 and 4), it can be inferred that the increase in butyrate does not depend on increased acetyl CoA in the context of inhibited methanogenesis but instead occurs via the direct stimulation of certain bacterial populations such as *Clostridia*. It remains unclear if the AT effect is due to bromoform or other compounds in AT that may be stimulatory, which may explain why the effect was significant in periods 1 and 2, while it is only marginal in periods 3 and 4, with an overall increase in butyrate formation. In contrast, we found that the gene copies (Fig. 2D) of acetate-CoA transferase (EC: 2.8.3.8) of the BP2 pathway increased in period 3, although not significantly, and is numerically higher in AT treatment compared to control. This is also in agreement with a significant increase in butyrate molar proportions in periods 1 and 2, but only marginal increases in periods 3 and 4. Therefore, the increase in butyrate synthesis as an alternate sink to inhibited methanogenesis by AT may not be accurate but may reflect a direct effect of HAT on butyrate-producing bacteria.

While there have been studies highlighting the potential of seaweeds, particularly AT (red seaweed) to significantly curb CH_4_ emission (60%–90%), risks associated with supply and side effects arising with increasing doses of AT have also been discussed (24, 28). Animal health concerns with feeding *A. taxiformis* should be considered. As discussed in reference (29), bromoform is categorized as a “potential human carcinogenic” compound by the EPA, and its impact on animal health is unclear (30). A summary of toxicological risk to animals and humans concluded that at low inclusion levels, *A. taxiformis* did not cause problems for ruminant animals or consumers through their products (24). In previous work, we conducted a milk sensory panel from a study where *A. taxiformis* was fed to dairy cattle (11), in which consumers were unable to distinguish milk from cows fed *A. taxiformis* from control milk, but the difference approached a trend (*P* = 0.11), with 39% of participants correctly identified milk from *A. taxiformis* cows as different from milk from control cows. Additionally, milk from cows fed *A. taxiformis* had five to eight times higher concentrations of iodine and bromide, respectively. Whereas the risk of bromide toxicity is unclear, the increase in iodine concentration in milk could have significant impacts on human health, particularly in persons with abnormal thyroid function. Given the high risk and high reward of AT in tackling methane mitigation from livestock globally, understanding the mechanistic basis of AT on ruminal methanogenesis, rumen microbial metabolism, and their collective long-term impact on animal health and productivity is pivotal to launching seaweeds as feed additive to livestock. This study provides the basis for the mode of action of AT, albeit transient, and provides essential insight for further investigation assessing inhibitor interactions with methanogens, the source of enteric CH_4_ formation in livestock.

## MATERIALS AND METHODS

### Animals and experimental design

The present study serves as an accompaniment to the animal investigation outlined in Stefenoni et al. (11). The detailed account of the animals and experimental design can be found in Stefenoni et al. (11). In summary, the study adopted a replicated 4 × 4 Latin square design, ensuring balance for residual effects. A cohort of 20 Holstein cows, comprising 4 primiparous and 16 multiparous individuals, with an average (±SD) of 2.6 ± 1.19 lactations, 95 ± 22.0 days in milk (DIM), and a starting milk yield of 42.2 ± 2.59 kg/day, were organized into five groups based on parity, DIM, and milk yield. The experimental design spanned four periods, each lasting 28 days, with a 21-day adaptation phase followed by a 7-day period for data and sample collection. Cows within each group were randomly assigned to one of the four treatments: control (basal diet without additives), 0.25% AT (LAT), 0.50% AT (HAT), or 1.77% O (oregano leaves). All cows were subjected to the same basal diet and received AT and O in a premix containing ground corn grain and wheat middlings, which was incorporated daily into the total mixed ration. The premix, stored at 4°C, was prepared biweekly, and cows received the full dose of AT and O from day 1 of each experimental period.

### DNA and RNA extraction, PCR amplification, and sequencing

The genomic DNA from solid ruminal samples was extracted using the repeated bead beating and column (RBB + C) method followed by extraction with the QIAmp Fast DNA Stool Mini Kit (Qiagen Sciences; Germantown, MD, USA) as described in reference (31). The extracted genomic DNA, both the V1–V2 regions of the bacterial 16S rRNA gene and the V6–V8 regions of the archaeal 16S rRNA gene, was PCR amplified in triplicate. The bacterial-specific primers used were F27 (5′-AGAGTTTGATCCTGGCTCAG-3′) and R338 (5′-TGCTGCCTCCCGTAGGAGT-3′), and the archaeal-specific primers used were i958aF (5′-AATTGGAKTCAACGCCKGR-3′) and i1378aR (5′-TGTGTGCAAGGAGCAGGGAC-3′). Both sets of primers were barcoded with a unique 12-base error-correcting Golay code for multiplexing as described in reference (32). Polymerase chain reaction was performed in triplicate using the Accuprime Taq DNA Polymerase System (Invitrogen). The thermal cycling conditions for PCR amplification of the bacterial 16S rRNA gene involved an initial denaturing step at 95°C for 5 min followed by 20 cycles (denaturing at 95°C for 30 s, annealing at 56°C for 30 s, extension at 72°C for 90 s) and a final extension step at 72°C for 8 min. The thermal cycling conditions for PCR amplification of the archaeal 16S rRNA gene involved an initial denaturing step at 94°C for 2 min followed by 30 cycles (denaturing at 94°C for 30 s, annealing at 56°C for 1 min 30 s, and extension at 72°C for 30 s) and a final extension step at 72°C for 8 min. The triplicate amplicon products from each sample were pooled and then quantified using a Spectramax M2e microplate reader (Molecular Devices, San Jose, CA, USA). The quantified amplicons were combined by adding each sample to a pool in equimolar concentration, and pools were bead purified using Agencourt AMPure XP Beads (Beckman-Coulter; Indianapolis, IN, USA). Sequencing was performed at the PennCHOP Microbiome Core, University of Pennsylvania, using the Illumina MiSeq platform. For metagenomics, DNA was prepared for whole-genome shotgun sequencing using the Nextera DNA Library Prep Kit (Illumina, San Diego, CA, USA). The library (tight insert size of 250 bp for high-throughput sequencing from both ends by 2 × 150 bp) was sequenced on an Illumina HiSeq 2500 at the Center for Host-Microbe Interactions at the University of Pennsylvania School of Veterinary Medicine.

### Bioinformatic analysis

The analysis utilized QIIME2 V2020.6 (33) for the processing of bacterial and archaeal 16S rRNA marker gene sequences. The initial steps involved demultiplexing the reads, followed by the truncation of forward and reverse reads at 230 nucleotides for bacteria and 220 nucleotides for archaea. Amplicon sequence variants were then generated through the utilization of the DADA2 plugin (34). Multiple sequence alignment was carried out using MAFFT (35), followed by the construction of a phylogenetic tree with FastTree 2 (36). Taxonomic assignments were derived by comparing ASV unique sequences to the Greengenes reference database v37 (37), via the naive Bayes classifier (38). Sample-to-sample diversity was evaluated by calculating weighted and unweighted UniFrac distances (39). For diversity analysis, the samples were rarefied to a count of 17,524 sequences for bacterial data sets and 1,377 sequences for archaeal data sets.

Shotgun metagenomics sequencing reads were quality filtered and trimmed using Trimmomatic v0.36 (40). Reads aligning to the cow host genome (ARS-UCD1.2/bosTau9) were removed using Bowtie2 (41). Taxonomic annotations were generated by Kraken2 (42) using the standard database with all complete bacterial, archaeal, and viral genomes in the National Center for Biotechnology Information’s RefSeq. Bracken v2.0 (43) was used to estimate the actual abundance of species in samples. To assess gene function and microbial pathways, reads were aligned to the Kyoto Encyclopedia of Genes and Genomes (KEGG) (44) database using DIAMOND v0.9.24 (45), and then the HUMAnN2 tiered search strategy (46) was used to quantify the KEGG Orthologous (KO) groups abundance against the KEGG database. Furthermore, the classification of hydrogenases was accomplished by aligning the quality-filtered reads to a hydrogenase database (47) through the utilization of the DIAMOND(45) search tool.

All statistical analyses were conducted using R version 4.2.1 (48) unless otherwise specified. Community-level differences in microbiomes across treatment groups were evaluated with the PERMANOVA test (49). Variations in 16S archaeal genera and metagenomic functional genes or enzymes among treatments were assessed through generalized linear models available in R stats package. The 16S bacterial genus data were analyzed using the SAS version 9.4 MIXED procedure (SAS Institute Inc., Cary, NC, USA), incorporating random effects for period and cow, fixed effects for treatment and period, and an interaction term for treatment and period. Period served as the repeated variable, with cow and treatment designated as subjects, and an autocorrelation structure of type “ar1” was employed as the covariance structure. Subsequently, least-squares means for “Treatment,” “Period,” and their interaction were computed.

In the analysis, relative abundances were employed for the assessment of archaeal genera, while CPM values were used for the evaluation of KEGG pathway genes and enzymes. For the analysis of bacterial genera, log-transformed values were applied. In instances of multiple tests, *P*-values were adjusted to control the false discovery rate, using the Benjamini-Hochberg method.

## Data Availability

The raw sequences obtained from 16S rRNA archaeal and bacterial sequencing, as well as metagenomics, have been archived in the NCBI Sequence Read Archive (SRA) database under the BioProject accession number PRJNA1084846.

## SUPPLEMENTAL MATERIAL

Data Set S1 mbio.00782-24-s0001.xlsx Abundant archaeal species (CPM) in methanogenesis pathways across treatment.

Data Set S2 mbio.00782-24-s0002.xlsx Effect of *Asparagopsis taxiformis* on hydrogenases (CPM) in metagenomics sequencing across treatments.

Data Set S3 mbio.00782-24-s0003.xlsx *Asparagopsis taxiformis* impact on bacterial composition in 16S rRNA sequencing across treatments.

Data Set S4 mbio.00782-24-s0004.xlsx *Asparagopsis taxiformis* impact on archaeal composition across treatments.

Supplemental material mbio.00782-24-s0005.docx Supplemental figures and data set legends.

ASM does not own the copyrights to Supplemental Material that may be linked to, or accessed through, an article. The authors have granted ASM a non-exclusive, world-wide license to publish the Supplemental Material files. Please contact the corresponding author directly for reuse.
