# Supplementary material for: Microbiome-informed study of the mechanistic basis of methane inhibition by Asparagopsis taxiformis in dairy cattle
Source: mBio. 2024 Jul 2;15(8):e00782-24. doi: 10.1128/mbio.00782-24 (PMC11323727; doi:10.1128/mbio.00782-24)
Supplement: Supplemental material — Supplemental figures and data set legends. [file mbio.00782-24-s0005.docx]

**Supporting Information for**

**Microbiome-informed study of the mechanistic basis of methane inhibition by *Asparagopsis taxiformis* in dairy cattle**

Nagaraju Indugu^1^, Kapil Narayan^1^, Hannah A Stefenoni^2^, Meagan L Hennessy^1^, Bonnie Vecchiarelli^1^, Joe Bender^1^, Reeti Shah^1^, Grace Dai^3^, Satvik Garapati^1^, Charles Yarish^4^, Sergio C Welchez^2^, Susanna E Räisänen^2^, Derek Wasson^2^, Camila Lage^2^, Andrea Melgar^2^, Alexander N Hristov^2^_,_ Dipti W Pitta^1*^

^1^Department of Clinical Studies-New Bolton Center, School of Veterinary Medicine, University of Pennsylvania, Kennett Square, PA 19348

^2^Department of Animal Science, The Pennsylvania State University, University Park, PA 16802

^3^Department of Computational Biology, School of Arts and Sciences, University of Pennsylvania, Philadelphia, PA 19104

^5^Department of Ecology and Evolutionary Biology, The University of Connecticut, Stamford 06901

*Corresponding author:

Dr. Dipti W. Pitta, dpitta@vet.upenn.edu

**This PDF file includes:**


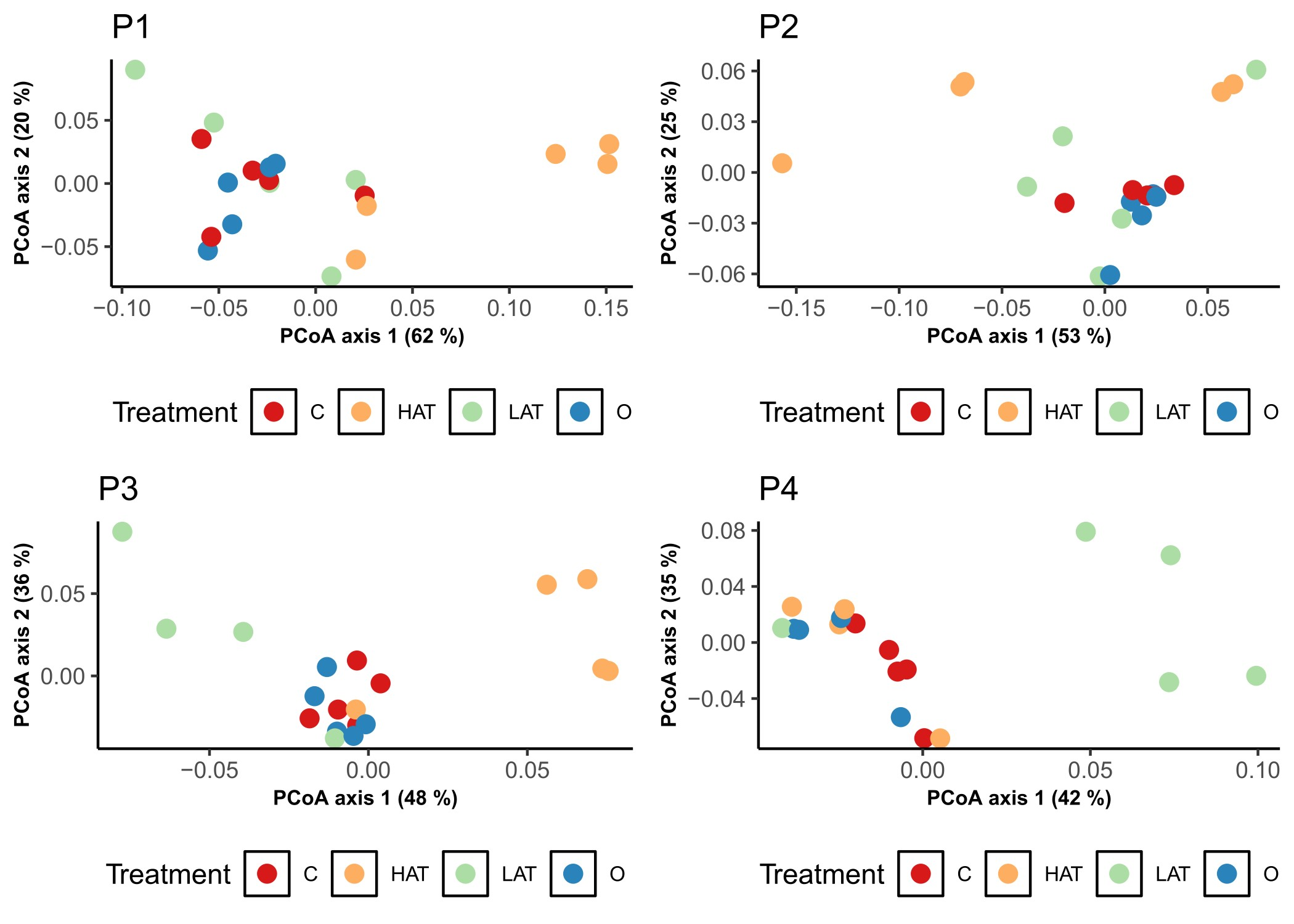


**Figure S1.** Principal Coordinates Analysis (PCoA) depicting unweighted UniFrac distances of 16S rRNA archaeal compositions in control, HAT (High dose of AT), LAT (Low dose of AT) and Oregano (O) treated cows across periods 1, 2, 3, and 4.


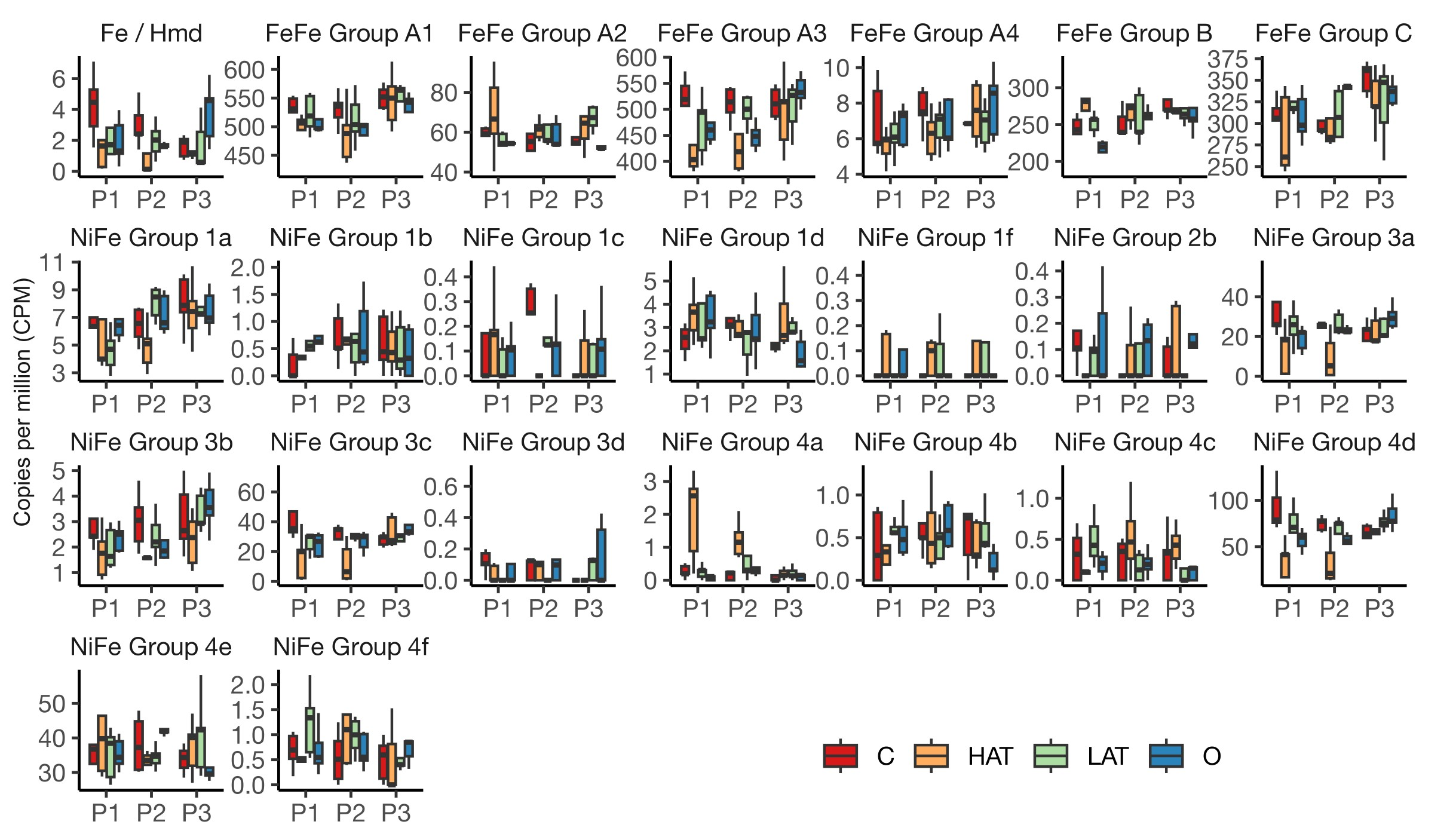


**Figure S2.** Comparison of gene abundance, measured in copies per million (CPM), associated with hydrogenase content across different treatments: Control (C), High Dose of AT (HAT), Low Dose of AT (LAT), and Oregano (O) treated cows. Hydrogenase content is shown based on hydrogenase subgroup. These are divided into fermentative hydrogenases (H_2_-producing; group A1, A2, B [FeFe]-hydrogenases), bifurcating hydrogenases (bidirectional; group A3, A4 [FeFe]-hydrogenases), respiratory hydrogenases (H_2_-uptake; group 1a, 1b, 1c, 1d, 1e, 1f, 1g, 1h, 1i, 1j [NiFe]-hydrogenases), respiratory hydrogenases (H_2_-evolving; group 4b, 4d [NiFe]-hydrogenases), alternative and sensory hydrogenases (H_2_-uptake; 2a, 2b [NiFe]-hydrogenases), cofactor-coupled bidirectional hydrogenases (3b, 3d, [NiFe]-hydrogenases), methanogenic hydrogenases (H_2_-uptake; group 1k, 3a, 3c, 4h, 4i [NiFe]-hydrogenases, [Fe]-hydrogenases), energy-converting hydrogenases (bidirectional; group 4a, 4c, 4e, 4f, 4g [NiFe]-hydrogenases), and sensory hydrogenases (group C [FeFe]-hydrogenases).


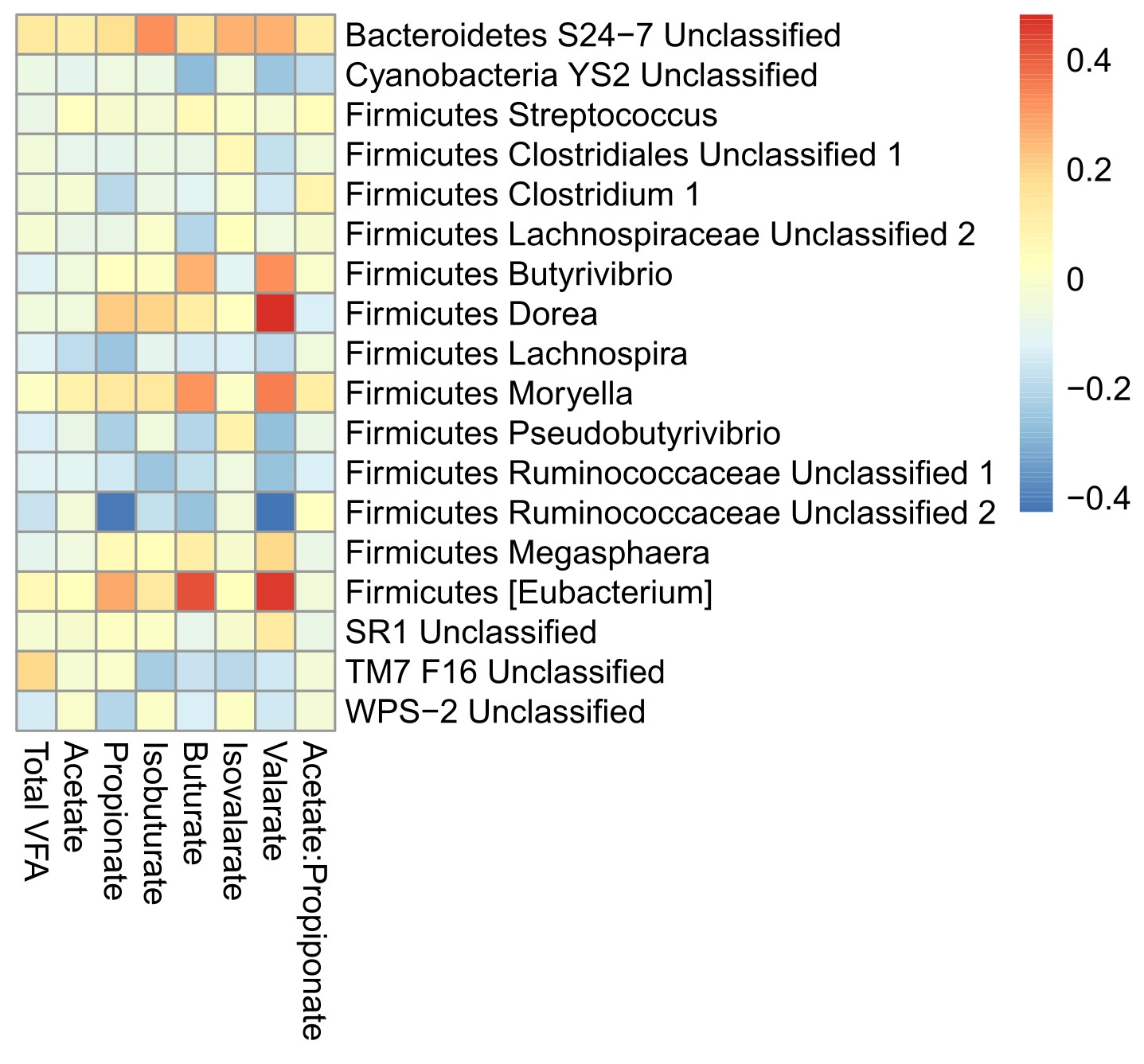


**Figure S3.** Associations between rumen bacteria and fermentation profiles based on Spearman correlation. Different colors of the bacterial genera show the corresponding phylum.; VFA, volatile fatty acids; mol%, molar proportion.


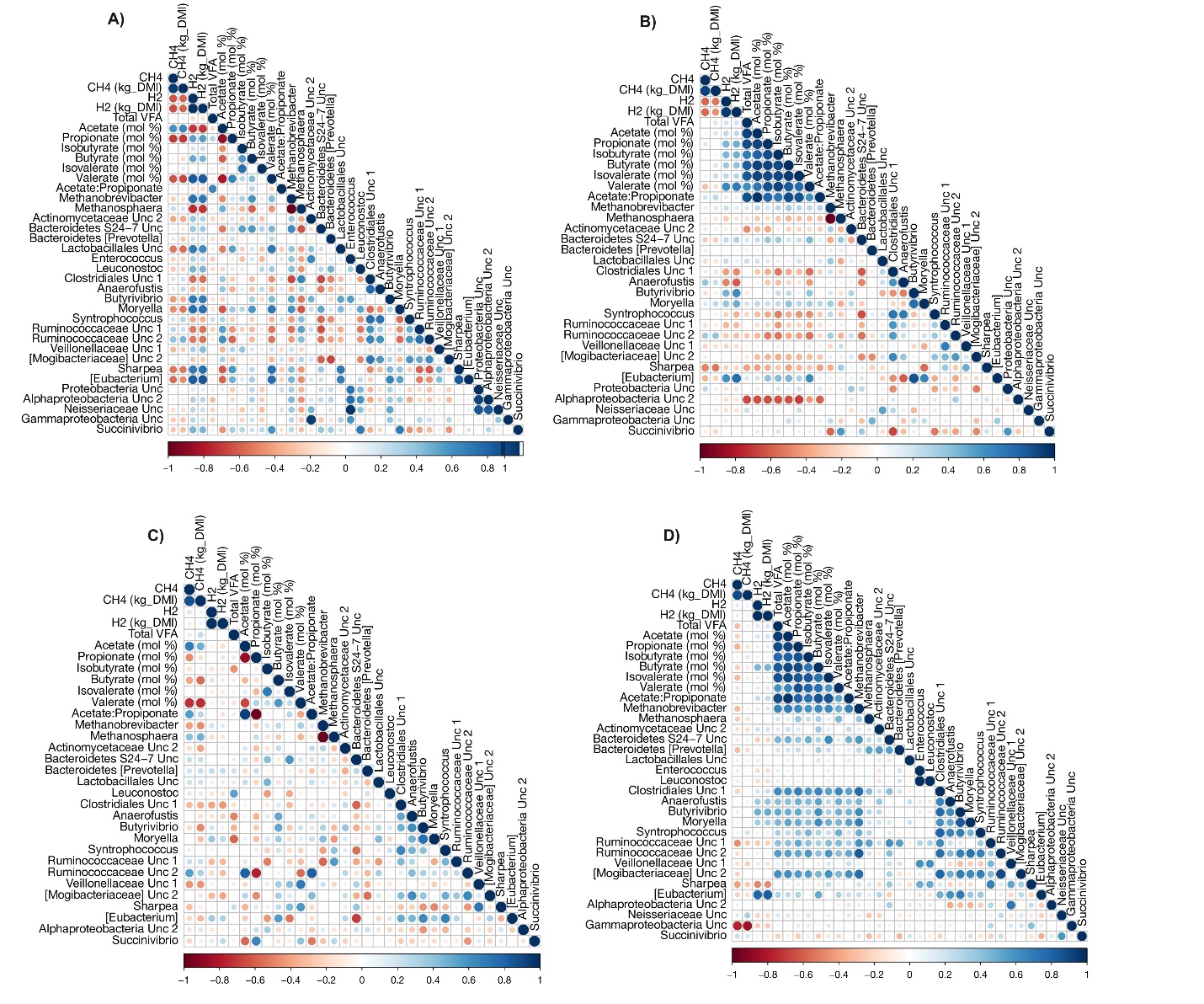


**Figure S4.** Associations between rumen bacteria and archaea and fermentation profiles using spearman correlation analysis by period, A) Period 1 B) Period 2 C) Period 3 and D) Period 4. Correlations are shown by the color code (Ligh blue to dark blue – moderate to high positive correlations; Light red to dark read- moderate to high negative correlations). CH_4_ methane; H_2_, hydrogen; VFA, volatile fatty acids; mol%, molar proportion.


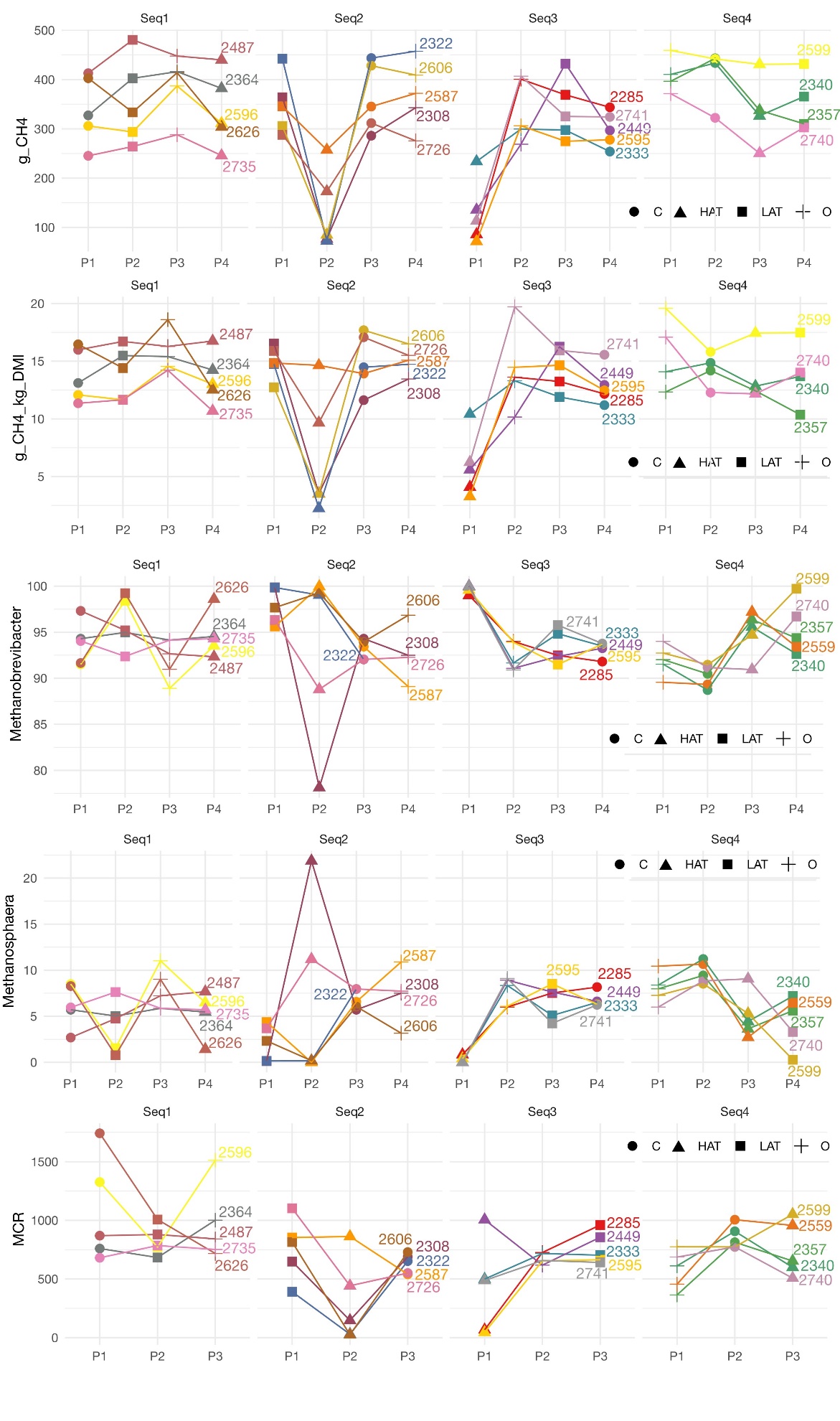


**Figure S5:** Sequence plots showing the effects of sequence (in which individual cows were rotated between treatment diets) on a) rumen methane production (gram/day), b) methane emissions (gram per kg dry matter intake), c & d) relative abundance of most abundant methanogens (*Methanobrevibacter*; *Methanosphaera*; %) and e) Methyl-coenzyme M reductase (MCR, copies per million). Twenty cows were assigned to four sequences with five cows per sequence. Each sequence was rotated in experimental periods over four weeks.

**Dataset S1**. The abundant archaeal species measured in copies per million (CPM), associated with methanogenesis enzymes across different treatments: Control (C), High Dose of AT (HAT), Low Dose of AT (LAT), and Oregano (O) treated cows. A) Carbon Dioxide (CO2)/Hydrogen (H_2_) methanogenesis pathway (KEGG pathway entry MD:M00567). B) Methanol Methanogenesis pathway (KEGG pathway entry MD:M00356). C, D, E) Methanogenesis pathway (KEGG pathway entry MD:M00563). F) Butyrate production from pyruvate to butyrate.

**Dataset S2.** Effect of *Asparagopsis taxiformis* on hydrogenases (cpm; copies per million) classified into H_2_ production and H_2_ consumption in metagenomics (metaG) sequencing across different treatments: Control (C), High Dose of AT (HAT), Low Dose of AT (LAT), and Oregano (O) treated cows.

**Dataset S3.** Effect of *Asparagopsis taxiformis* on bacterial taxonomical composition (relative abundance %) in DNA-based 16S rRNA sequencing across different treatments: Control (C), High Dose of AT (HAT), Low Dose of AT (LAT), and Oregano (O) treated cows. A) Phylum B) Genus. SEM: Standard error of mean.

**Dataset S4**. Effect of *Asparagopsis taxiformis* on archaeal taxonomical composition (absolute abundance) across different treatments: Control (C), High Dose of AT (HAT), Low Dose of AT (LAT), and Oregano (O) treated cows. A) 16 rRNA genus level B) metagenomics at species level.
